# Some Points about the Meeting at Omaha

**Published:** 1898-11

**Authors:** 


### SOME POINTS ABOUT THE MEETING AT OMAHA.

President Salmon was strongly averse to a definite period of office for the President, and urged its remaining open to the will and decision of the members.

Much is claimed, a great deal is hoped for, by those who urged a reduction of the dues to three dollars per year. A large increase of membership was promised, and a more prompt payment of dues assured. Time will tell how great the results.

Dr. R. R. Bell, of Brooklyn, N. Y., advocated greater facilities for clinical work and demonstrations.

The Committee on Diseases for the coming year will specially direct their investigations and prepare statistics of osteoporosis and rabies.

Dr. A. H. Baker, of Chicago, strongly advocated the election of Dr. Clement as President, and ably seconded the nomination.

Quite an interesting vote was recorded on the central vice-presidency, Dr. A. H. Baker, of Chicago, receiving twenty-two and Dr. John R. Mitchell, of Evansville, Ind., eighteen.

Those advocating an informal ballot did not wish to place their favorite candidates in nomination.

Prof. Law, in discussing the report of the Committee on Intelligence and Education, warmly complimented the tenor of the report on the course of education outlined by Chairman Pearson.

The very practical paper by Dr. R. R. Bell, on "Acute Indigestion of the Horse," was freely discussed by Drs. Whitbeck, Williams, Merillat, Cary, Nelson, Walrod, Clement, Nighbert, A. H. Baker, and C. C. Lyford.

President Clement, on taking the chair, was brief in his remarks, but deeply appreciative of the honor conferred upon him.

Secretary Stewart, on again renewing his devotions to the duties of that office, thanked the members for their assistance in the past.

The extent and outlines of the arytenoid cartilage was a hard bone of contention among those who were frequent operators on "Roarers."

